# Supplementary material for: New Insights into the Chemical Reactivity of Dry-Cured Fermented Sausages: Focus on Nitrosation, Nitrosylation and Oxidation
Source: Foods. 2021 Apr 14;10(4):852. doi: 10.3390/foods10040852 (PMC8070781; doi:10.3390/foods10040852)
Supplement: Supplementary file 1 [file foods-10-00852-s001.pdf]

Table S1: Effect of added doses of sodium nitrite and nitrate on the concentration of residual nitrite, residual nitrate, nitrosothiols and nitrosamines in dry-cured fermented sausages.

	Conditions of nitrite / nitrate added			
	0 NO_2/NO_3	80 NO_2/NO_3	200 NO_3	120 NO_2/NO_3
Nitrite (µM)	3.6^{a}	79.1^{b}	96.0^{b}	183.3^{c}
Nitrate (µM)	67.6^{x}	110.5^{x}	211.9^{y}	343.5^{y}
Nitrosothiols (µM)	5.7^{α}	0.4^{α}	11.5^{α}	34.3^{α}
Nitrosamines (µM)	ND	ND	204.0^{β}	123.0^{β}

The concentrations of their components are expressed in µM. Values are mean of 6 independent determinations. Values without common superscripts, a, b, c for nitrite, x, y, z for nitrate, α for nitrosothiols and β for nitrosamines differ significantly ($p < 0.01$). ND for not detected.

Table S2: Molecular interactions in cured and dry fermented sausages studied by correlation matrix.

	Free iron	Nitrosylheme	Heme iron	TBARS	Free thiols	Carbonyls	Nitrite residuals	Nitrate residuals	Nitrosothiols	Nitrosamines	Proteolysis index
Free iron											
Nitrosylheme	-0,67										
	p=0,0001										
Heme iron	0,72	0,80									
	p=0,0001	p=0,0001									
TBARS	0,68	-0,88	-0,72								
	p=0,0001	p=0,0001	p=0,0001								
Free thiols	0,15	-0,33	-0,19	0,24							
	p=0,479	p=0,112	p=0,386	p=0,256							
Carbonyls	-0,30	0,59	0,63	-0,40	-0,15						
	p=1,555	p=0,002	p=0,001	p=0,052	p=0,477						
Nitrite residuals	-0,60	0,78	0,72	-0,74	-0,37	0,57					
	p=0,002	p=0,0001	p=0,0001	p=0,0001	p=0,073	p=0,003					
Nitrate residuals	-0,41	0,63	0,60	-0,62	-0,47	0,34	0,83				
	p=0,044	p=0,001	p=0,002	p=0,001	p=0,021	p=0,104	p=0,0001				
Nitrosothiols	-0,10	0,43	0,16	-0,37	-0,42	0,09	0,31	0,62			
	p=0,629	p=0,037	p=0,457	p=0,076	p=0,040	p=0,682	p=0,135	p=0,001			
Nitrosamines	-0,23	0,20	0,34	-0,33	0,05	0,07	0,28	0,35	0,07		
	p=0,279	p=0,360	p=0,105	p=0,121	p=0,819	p=0,752	p=0,178	p=0,093	p=0,759		
Proteolysis index	0,49	-0,83	-0,80	0,61	0,16	-0,65	-0,62	-0,40	-0,08	0,04	
	p=0,016	p=0,0001	p=0,0001	p=0,002	p=0,452	p=0,001	p=0,001	p=0,054	p=0,709	p=0,855	
